# Impact of dental visiting patterns on oral health: A systematic review of longitudinal studies

**DOI:** 10.1038/s41405-024-00195-7

**Published:** 2024-03-06

**Authors:** Aina Najwa Mohd Khairuddin, Birke Bogale, Jing Kang, Jennifer E. Gallagher

**Affiliations:** 1 Dental Public Health, Centre for Host-Microbiome Interactions, Faculty of Dentistry, Oral and Craniofacial Sciences, King’s College London, London, UK; 2 Department of Community Oral Health & Clinical Prevention, Faculty of Dentistry, Universiti Malaya, Kuala Lumpur, Malaysia; 3 Department of Dental and Maxillofacial Surgery, St Paul’s Hospital Millennium Medical College, Addis Ababa, Ethiopia; 4 Oral Clinical Research Unit, Faculty of Dentistry, Oral and Craniofacial Sciences, King’s College London, London, UK; 5 Oral Biology, School of Dentistry, University of Leeds, Leeds, UK

**Keywords:** Dental epidemiology, Oral diseases

## Abstract

**Aim:**

To systematically review longitudinal studies investigating the impact of dental visiting patterns on oral health across the life course.

**Methods:**

Five databases (MEDLINE, Embase, Scopus, Web of Science, CINAHL) were searched up to March 2023. Results were screened based on eligibility criteria in a two-stage process: title and abstract, and full-text review. A backward search of reference lists and a forward search of citations of the included papers was also conducted. The quality of the included papers was assessed using the Newcastle-Ottawa Scale. Key study information was extracted and a narrative synthesis of the findings was performed.

**Results:**

Eleven papers from five longitudinal studies in five countries (Australia, Brazil, China, New Zealand, Sweden) met the inclusion criteria. Studies of moderate to high quality consistently reported that regular dental attendance was associated with having less dental caries experience, fewer missing teeth and better oral health-related quality of life. Inconsistent findings were observed for decayed teeth, and no association was found for periodontal condition.

**Conclusions:**

This review highlights an association between regular dental visiting pattern and improved oral health, notably less dental caries experience and better oral health-related quality of life. Dental attendance emerges as an important predictor of oral health across the life course, underscoring the importance of routine dental care.

**Registration information:**

The PROSPERO registration number is CRD42023396380.

## Introduction

Dental diseases burden billions of people worldwide [1] with pain, discomfort, functional impairment and impaired quality of life [2]. Acknowledging the importance of having access to dental care for the prevention and treatment of oral diseases, the World Health Organization (WHO) Global Strategy on Oral Health advocates for universal health coverage to ensure equitable access to essential dental services [3]. The World Health Assembly has established a goal of ensuring 80% of people worldwide have access to essential oral healthcare services by the year 2030 and outlined the Global Oral Health Action Plan [4]. Despite this advocacy, disparities in the utilisation of oral healthcare persist globally. These disparities are influenced by factors such as a country’s development status, family structure, health literacy, general health status and healthcare costs [5, 6].

While healthcare system characteristics and social factors play significant roles in determining access to care, behavioural factors are equally influential, as outlined in Andersen et al.’s model of access to healthcare [7]. There is extensive evidence on oral health inequalities attributed to oral health-related behaviours, such as oral hygiene, smoking, dietary practices and dental attendance. Recent reviews using the life course framework suggested that early life exposures, including access to dental care, have long-term consequences for oral health in adulthood [8, 9]. Various factors contribute to the dynamics of this association. For example, in children, dental attendance can be affected by their social class and their mothers’ dental attendance patterns [8–10]. In adults, influences include plaque levels, the presence of calculus, anxiety levels, and oral health-related preventive behaviours such as the frequency of toothbrushing and the use of additional dental hygiene products [11]. Additionally, lower utilisation of dental services is noticeable among younger children, individuals with edentulism, severe tooth loss, poor health literacy, as well as those with general and oral health issues [5]. However, this evidence is mostly from cross-sectional studies where dental attendance was treated as one of the covariates in the analysis, rather than as the main predictor.

Several decades ago, there were critiques of regular dental attendance [12, 13]. A review in 1977 suggested that a standard 6-month recall interval may not be necessary, considering the slow rate of dental caries progression, decreased caries activity with age, and the presence of fluoride in water [12]. This review further suggested that extending the interval between examinations may reduce unnecessary treatment [12]. Additionally, a cross-sectional study in 1985 revealed that regular dental attenders, particularly those who visit dentists every 6 months, had a higher number of filled teeth and thus, higher caries experience than those attending only when in trouble [13]. The authors of this study suggested that regular attendance is suitable only to prevent tooth loss and maintain dental function, but not effective in preventing further dental caries or disease. However, with the progression of time and research, as well as the increasing reorientation of dental care towards prevention [14], dental professionals’ advocacy for regular dental visits has been substantiated by a growing body of evidence. Given the preventability of most dental diseases, contemporary science thus supports risk-based recall intervals, as recommended by the National Institute for Health and Care Excellence (NICE) [15, 16].

A systematic review, consisting of mainly cross-sectional studies, suggested that routine dental visits is associated with positive impacts on oral health – higher number of remaining teeth and better perceived oral health [17]. Epidemiological evidence, based on cross-sectional studies, indicated that symptomatic dental attenders tend to have poorer oral health than those who adhere to routine dental visits, as demonstrated by greater caries experience, and more decayed and missing teeth [11, 17]. To better explore the impact of oral healthcare utilisation on oral health over time, longitudinal studies, which follow individuals from early life onwards, can offer valuable insights. For example, the Dunedin Multidisciplinary Health and Development Study, following a birth cohort from 1972/73, found that routine attenders had better self-reported oral health, less tooth loss and less dental caries than their counterparts [18]. If well conducted, this study design is known to provide a helpful time sequence of events and tracking intragenerational changes over time, and is ideal for testing causal life course hypotheses [19]. However, to date, there has been no systematic review that brings together the body of evidence from longitudinal studies. Thus, this review aims to systematically identify and synthesise findings from longitudinal studies exploring the impact of dental visiting patterns on oral health across the life course. A collective analysis of the findings will be beneficial for public health measures in support of the WHO global strategy for oral health.

## Methods

The protocol for this review has been registered in the International Prospective Register of Systematic Reviews (PROSPERO) (CRD42023396380). The reporting followed the Preferred Reporting Items for Systematic Reviews and Meta-Analyses (PRISMA) guidelines [20].

### Eligibility Criteria

The inclusion and exclusion criteria were as follows:

Inclusion criteria

1. Population: Individuals of any age and gender.
2. Exposure: Dental visiting patterns or use of oral healthcare services recorded at baseline or more than one time points.
3. Outcome: Any oral health-related outcome, either diagnosed clinically or self-reported.
4. Study design: Longitudinal studies.

Exclusion criteria

1. Articles with abstract/full text that are not available in English.
2. Experimental studies, cross-sectional studies, case-control studies, retrospective studies, case reports and reviews.
3. Articles not related to the dental field.

### Information sources

A comprehensive search was conducted in five databases – MEDLINE and Embase via Ovid, Web of Science, Scopus, and CINAHL – from their inception until March 2023. To ensure a thorough review, forward and reverse citations searching was performed for all included papers. No restrictions were imposed on language or publication year during the search process.

### Search strategy

The search strategy was designed based on three key concepts: (1) life course; (2) dental visit; and, (3) oral health. To capture the breadth of relevant literature, a combination of Medical Subject Headings (MeSH) and keywords were adapted for each of the databases (see File S1).

### Study selection

Search results from the databases were collated using EndNote 20 [21], and duplicate references were removed. The remaining references were exported to Rayyan [22], an online screening tool, which facilitated a semi-automated screening process. Two reviewers (ANMK, BB) independently conducted the initial screening of titles and abstracts manually using Rayyan. Following this, blind comparison of include/exclude/undecided decisions was automatically generated. Full text of the ‘include’ and ‘undecided’ abstracts were then retrieved for the final screening, adhering to the eligibility criteria. Any discrepancies in decisions were discussed with two additional reviewers (JEG, JK) until consensus was achieved.

### Data collection

A data extraction form was created using Microsoft Excel to systematically capture key study information. This form encompassed details such as population setting, data source, sample size, study methods, follow-up period, dental visiting patterns, oral health measures and study results. Data extraction was conducted by the lead author (ANMK), and the accuracy of the extracted data was verified by JEG and JK through cross-checking.

### Quality assessment

Two reviewers (ANMK, BB) independently evaluated the included papers for risk of bias using the Newcastle-Ottawa Scales (NOS) (Table S1) [23]. The NOS tool provides a straightforward scale for an overall quality assessment and is convenient to use for reporting the quality of primary papers in systematic reviews [24]. Its applicability has been demonstrated in prior systematic review of longitudinal studies [25]. To assess the quality of the selected papers, the NOS was applied, with a point awarded for each starred response. The evaluation covered three main domains with a possible total score of 9 points: selection (4 points), comparability (2 points) and outcome (3 points) [23]. Quality ratings ranged from 1 (very poor) to 9 (high), with scores less than 5 indicating low quality, scores between 5 and 7 indicating moderate quality, and scores above 7 indicating high quality [17, 26]. All papers meeting the selection criteria were included, regardless of quality. In cases of scoring discrepancies, resolution was achieved via discussions with JEG and JK to reach a consensus.

### Data synthesis

Based on the extracted data, meta-analysis was deemed inappropriate due to marked heterogeneity among studies mainly from apparent differences in study population (age and phase in life course), settings (frequency and duration of follow-up), categorisation of dental visiting patterns and oral health outcome measures. The findings were classified based on the nature of oral health outcomes: clinical (total dental caries experience, periodontal problem) and self-reported (tooth loss, general oral health status, oral impact on daily performance, etc.). A narrative synthesis of the findings was employed, aligning with the recommendation by Campbell et al. [27]. Within this approach, similarities and differences observed in the findings were highlighted.

## Results

### Study selection

**Fig. 1:**
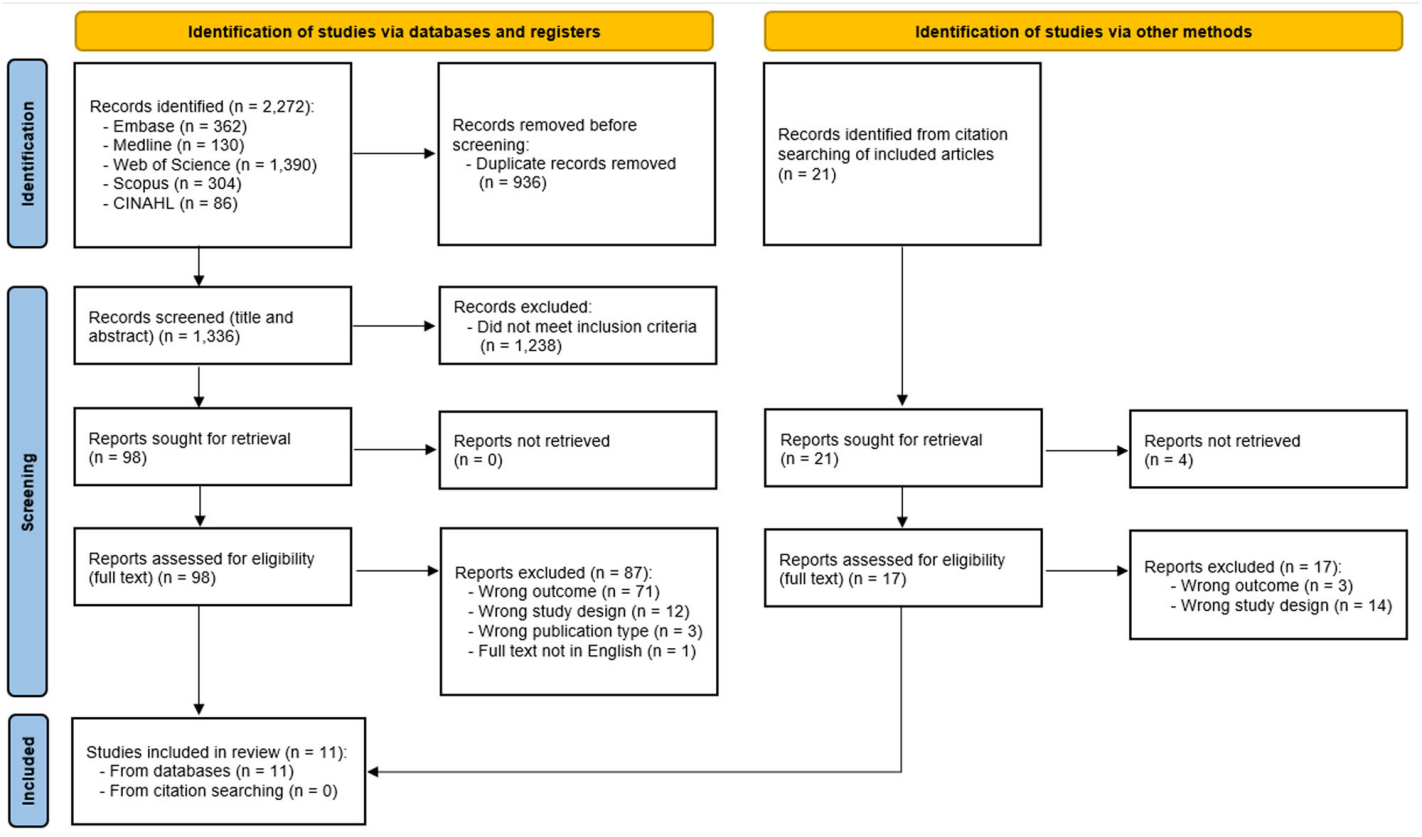
PRISMA flowchart. A diagram demostrating the process of identifying, screening, and including papers in this review.

The search across five electronic databases yielded a total of 2,272 papers. After removing duplicates, 1,336 papers remained for the initial screening of titles and abstracts. Of these, 98 progressed to full-text screening. An additional 21 papers, identified through forward and backward searching, were included in the screening process. Overall, 11 papers met the eligibility criteria and were accepted for inclusion in the review [18, 28–37]. The screening process is summarised in the PRISMA flowchart (Fig. 1).

### Study characteristics

In this review, the term ‘study’ is used to denote a longitudinal study that gathers primary information, and the term ‘paper’ refers to a publication arising from primary or secondary research, utilising data from a longitudinal study to address a specific research question. The 11 papers included in this review were derived from five longitudinal studies, conducted in Australia [31], Brazil [36], Hong Kong [30], New Zealand [18, 32, 34, 37] and Sweden [28, 29, 33, 35]. The summarised characteristics of each included paper are presented in Table S2. The scope of the longitudinal studies included in this review was broad, encompassing various study populations across the life course. Participants ranged in age from 12 months to 70 years across all studies, with follow-up durations ranging from 12 months to 20 years. One paper focused solely on childhood participants [36], another one focused only on adolescence [30], while two focused exclusively on adults [34, 37]. Three papers followed the cohorts from adolescence to adulthood [18, 31, 32], and four investigated cohorts from adulthood to elderhood [28, 29, 33, 35]. Data collection for dental attendance predominantly relied on self-completed questionnaires (*n* = 10), whilst a paper from the Australian study [31] utilised service-use logbooks and dental treatment audits. As for oral health outcomes, the majority of the papers relied on self-reported responses, [28, 29, 31, 33, 35–37] with just four assessing the outcomes via clinical examinations [18, 30, 32, 34].

The definition and categorisation of dental attendance varied among the included papers. Six papers took into account both the duration and purpose of dental visits to categorise exposure groups into routine and nonroutine attendance [18, 32, 33, 35–37]. Four papers categorised dental attendance based only on duration [28–31], and one [34] solely considered the reason for dental visits––whether for a check-up (regular attenders) or only when a dental problem occurred (nonregular attenders). Most papers (*n* = 9) considered a dental visit within 12 months as ‘regular attendance’, although one reported a shorter duration of 6 months [36], and another one considered a dental visit within 3 years [30]. In addition, the number of comparison groups differed between the included papers; most reported two categories of dental attendance, with only two papers [32, 37] reporting three categories (see Table 1). Some papers further derived and categorised dental attendance patterns into four groups; for example, ‘stable annual’, ‘annual - not annual (downward)’, ‘not annual - annual (upward)’, ‘stable not annual’ [28], and ‘nonroutine’, ‘routine - nonroutine’, ‘nonroutine - routine’, ‘routine’ [33, 35, 36].

**Table 1:** Categorisation of dental attendance.

Paper	Nature of category	Number of groups	Name of category	Definition
Åstrøm et al. (2011) [28], Astrom et al. (2011) [29], Crocombe et al. (2012) [31]	Duration of visit only	2	Regular attendance	Had a dental visit in the past 12 months.
			Nonregular attendance	Never had a dental visit in the past 12 months.
Lu et al. (2011) [30]	Duration of visit only	2	Dental attendance: yes	Had dental visits during the age of 12–15 years and during the age of 15–18 years.
			Dental attendance: no	Never had dental visits during the age of 12–15 years and during the age of 15–18 years.
Broadbent et al. (2016) [34]	Purpose of visit only	2	Regular attendance	Usually attends for check-ups.
			Nonregular attendance	Usually attends due to dental problems.
Menegazzo et al. (2020) [36]	Duration and purpose of visit	2	Routine attendance	Had a dental visit in the past 6 months for a check-up.
			Nonroutine attendance	Never had a dental visit in the past 6 months or attends due to a dental problem.
Åstrøm et al. (2014) [33], Åstrøm et al. (2018) [35], Thomson et al. (2010) [18]	Duration and purpose of visit	2	Routine attendance	Had a dental visit in the past 12 months and initiated by the dentist / for a check-up.
			Nonroutine attendance	Never had a dental visit in the past 12 months and visited due to a dental problem.
Crocombe et al. (2012) [32]	Duration and purpose of visit	3	Regular attenders	Had a dental visit in the past 12 months and usually for a check-up in all 4 sweeps.
			Opportunists	Had a dental visit in the past 12 months in 2 sweeps, but in later sweeps visited only due to dental problems.
			Decliners	Had a dental visit in the past 12 months in 1 sweep, but in later sweeps visited only due to dental problems.
Hong et al. (2023) [37]	Duration and purpose of visit	3	Always routine	Had a dental visit in the past 12 months and usually for a check-up.
			Sometimes routine	Other rest of participants.
			Never routine	Never had a dental visit in the past 12 months and usually due to dental problems.

### Quality assessment

The included papers received a moderate quality rating, except for one, by Crocombe et al. [32] which had high quality rating based on the NOS checklist (Table 2). The paper by Crocombe et al. [32] demonstrated a robust methodology, characterised by an extended duration of follow-up, minimal attrition, assessment of outcomes through both clinical evaluation and self-report, and rigorous control for both common and additional confounders. Notably, almost all papers (*n* = 10) did not meet the criteria for a robust method of ascertaining exposure, primarily relying on self-reported information. Similarly, for outcome assessment, seven papers relied on self-reported oral health outcomes rather than clinical evaluation. About half of the included papers (*n* = 6) did not consider potential confounders such as dental anxiety, accessibility and cost [18, 30, 34–37].

**Table 2:** Quality assessment of included papers.

No.	Paper	Selection				Comparability	Outcome			Total score (1✩ = 1 point)	*Paper quality
		Representativeness of the exposed cohort	Selection of the non-exposed cohort	Ascertainment of exposure	Outcome of interest not present at start of study	Controls for confounders	Method of assessment	Sufficient duration of follow-up	Adequacy of cohorts follow-up		
1	Åstrøm et al. [28].	✩	✩	/	✩	✩✩	/	✩	/	6	Moderate
2	Åstrøm et al. [29].	✩	✩	/	✩	✩✩	/	✩	/	6	Moderate
3	Åstrøm et al. [33].	✩	✩	/	✩	✩✩	/	✩	/	6	Moderate
4	Åstrøm et al. [35].	✩	✩	/	✩	✩	/	✩	/	5	Moderate
5	Thomson et al. [18].	✩	✩	/	✩	✩	✩	✩	✩	7	Moderate
6	Crocombe et al. [32].	✩	✩	/	✩	✩✩	✩	✩	✩	8	High
7	Broadbent et al. [34].	✩	✩	/	✩	✩	✩	✩	✩	7	Moderate
8	Hong et al. [37].	✩	✩	/	✩	✩	/	✩	✩	6	Moderate
9	Crocombe et al. [31].	✩	✩	✩	✩	✩✩	/	/	/	6	Moderate
10	Lu et al. [30].	✩	✩	/	✩	✩	✩	✩	/	6	Moderate
11	Menegazzo et al. [36].	✩	✩	/	✩	✩	/	✩	/	5	Moderate

* Total score < 5 = low quality; 5 to 7 = moderate quality; > 7 = high quality

In the Swedish longitudinal study that tracked participants for 15 years, only 55% to 65% of the participants responded at certain sweeps, and they were more likely to be of the native population [33], married civil status [33, 35], perceived good health [33] and routine dental attenders [33, 35], while non-respondents were more likely to be smokers, have lower education, be unemployed and have fewer teeth [28, 29, 35]. Consequently, this led to an overrepresentation of women and participants with higher education [28, 29, 35]. Across the five longitudinal studies, only the Dunedin study (four papers, *n* = 4) [18, 32, 34, 37] had a follow-up rate of more than 80%, whilst the other four studies (seven papers, *n* = 7) [28–31, 33, 35, 36] reported attrition rates exceeding 20%. This could further introduce selection bias due to loss of follow-up [38] and compromise the generalisability of study findings to the population [39].

### Dental caries experience

Four papers from two longitudinal studies (Hong Kong and Dunedin) reported clinically measured outcomes pertaining to dental caries experience in permanent dentition, which includes total decayed, missing and filled surfaces or teeth (DMFS or DMFT), as well as each component separately [18, 30, 32, 34]. The quality of these papers ranged from moderate to high (NOS = 6 to 8). As age increased, the mean DMFT score showed a gradual rise, and dental service utilisation was found to have a direct positive impact on the overall dental caries experience [30]. A similar trend was observed for the mean DMFS score, whereby routine dental attenders exhibited a significantly lower mean DMFS score than their nonroutine counterparts [18]. Crocombe et al. [32] also reported that opportunistic dental attenders had a higher mean DMFS than the regular attenders; however, no significant association was found between regular dental attenders and those who declined dental appointments [32]. In terms of untreated decayed tooth surfaces (DS), two papers showed that regular dental attenders had a lower mean number of DS than their counterparts [18, 34]. Conversely, one paper by Crocombe et al. [32] reported no significant associations between dental visiting patterns and DS and filled tooth surfaces. Additionally, a lower likelihood of missing teeth due to dental caries was observed among routine/regular attenders than nonroutine/opportunistic dental attenders [18, 32, 34].

### Periodontal condition

Periodontal health status was clinically assessed only by Lu et al. [30] from the Hong Kong longitudinal study. This paper was of moderate quality (NOS = 6), primarily due to the low follow-up rate (50.8%) and potential recall bias. In the extended path analysis, the authors observed rising levels of periodontal disease from childhood to adolescence, regardless of attendance patterns; however, dental service utilisation did not have a significant influence on periodontal health status.

### Tooth loss

Two studies of moderate quality (NOS = 6), derived from the Swedish longitudinal study, examined self-reported tooth loss [29, 33]. Despite originating from the same data source, these papers categorised both the independent and dependent variables differently, leading to some contrasting results. The paper by Åstrøm et al. [29] reported a significant increase in the prevalence of tooth loss as age increases and found no significant association between dental care utilisation and tooth loss patterns (‘stable tooth loss’, ‘change from all teeth maintained to tooth loss’, ‘all teeth maintained’). However, absence from dental visits particularly due to financial constraints was significantly associated with unfavourable tooth loss patterns. In contrast, in the more recent paper, the authors found that long-term nonroutine dental care utilisation was significantly associated with major tooth loss (‘lost many or all teeth’) [33].

### Oral health-related quality of life

Self-reported oral health-related quality of life (OHRQoL) emerged as the most frequently examined outcome measure, investigated in seven of the included papers from four longitudinal studies (Sweden, Dunedin, Australia and Brazil) [28, 31–33, 35–37]. The quality of evidence ranged from moderate to high quality (NOS = 5 to 8). Only one study from Brazil [36] evaluated OHRQoL among children, using the Child Perception Questionnaire (CPQ8-10) [40]. The authors found that routine dental attenders demonstrated lower mean CPQ8-10 scores, indicating fewer oral symptoms and functional limitations, as well as better emotional and social well-being, than those with nonroutine dental attendance. The Swedish study focused on elderly people, utilising the Oral Impacts on Daily Performance (OIDP) [41] instrument. From the study, two papers found that long-term annual/routine dental attenders were significantly less likely to experience oral impacts (i.e., daily performance affected) than nonannual/nonroutine attenders [28, 33]. One other paper from the same study reported changes in OIDP scores as the outcome measure and found that long-term nonroutine dental attenders were more likely to experience improvements in OIDP than routine attenders [35]. Similar finding was observed for worsened OIDP [35]. Three papers from the Dunedin [32, 37] and Australian [31] studies utilised the Oral Health Impact Profile (OHIP-14) [42] to measure OHRQoL. Those who had never accessed routine dental care or only visited due to dental problems throughout adulthood demonstrated higher mean OHIP-14 scores than those who consistently attended routine dental care [32, 37]. Furthermore, a stratified analysis based on residential location revealed a positive association between dental attendance and improvement in OHIP-14 scores among people residing outside the capital city [31].

### General oral health status

Two papers of moderate to high quality from the Dunedin study evaluated self-perceived oral health [18, 32]. These papers consistently showed that routine dental attenders reported better oral health status than those with less-favourable visiting patterns.

## Discussion

This systematic review examined data across 11 papers from five longitudinal studies, investigating the impact of dental visiting patterns on oral health. Four longitudinal studies were from high-income countries and one from upper middle-income countries. Evidence of moderate to high quality revealed that regular dental attendance had a positive impact on clinical and self-reported oral health outcomes, particularly less dental caries experience and fewer missing teeth, [18, 29, 30, 32–34] better OHRQoL, [28, 31, 33, 35–37] and better self-rated oral health status [18, 32].

A widely applied conceptual model in oral health-related studies, Andersen’s Behavioural Model of Health Services Use (fourth version) [7] may plausibly explain the findings of the included papers in our review. The disparities in both dental care utilisation and oral health outcomes are contributed by predisposing factors, such as education, income, occupation and social class [18, 31, 32, 34–36]. A review on the impact of socioeconomic gradient on oral health also highlighted that low socioeconomic status limits oral healthcare utilisation, thereby contributing to social discrepancies in oral health status [26]. Besides that, the utilisation of dental services and the status of oral health are influenced by enabling resources, such as accessibility, treatment cost and the type of oral healthcare [28, 29, 31, 33]. This carries significant implications, particularly for countries grappling with limited access to oral healthcare systems [5]. Difficulties in accessing dental care may also arise from avoidance of visiting the dentist due to high treatment costs and financial constraints [6, 43], as well as reduced mobility due to physical and mental disability, particularly among the elderly population [44, 45]. In such contexts, the lack of regular dental check-ups may exacerbate oral health problems, contributing to broader health issues. Additionally, given the socioeconomic differences, advantaged people may exhibit favourable attendance patterns and seek private dental clinics for more complex, tooth-saving, albeit expensive treatments; while disadvantaged people tend to display symptomatic attendance patterns and seek public dental care which is much more affordable [31].

Oral-health-related behaviours play an important role in dental visiting patterns and oral health status [18, 28, 29, 32, 34, 35, 37]. As postulated by Alexander et al. [46], symptomatic dental attenders may have distinct philosophical orientations concerning the importance of preventive care than the regular dental attenders. The dissimilarities in oral health status between regular and nonregular attenders may be attributed to the phenomenon known as the “healthy user effect” [47], an effect encapsulating a cluster of behaviours conducive to better health outcomes, such as health-conscious, avoidance of smoking, moderate alcohol consumption, prudent dietary and hygiene habits, as well as routine healthcare visits and health screenings. This corroborates our findings that people with frequent toothbrushing [34, 37] and having lower plaque scores [18, 32], exhibit better oral health outcomes, indicative of the influence of the healthy user effect. However, a study by Listl et al. [48] which evaluated data from 13 European countries, suggested that the relationship between dental visiting patterns and oral health status is not merely attributable to the healthy user effect, but is indeed causal. Besides the above, the association between dental anxiety and oral health should also be noted. One of the included papers found that dentally anxious people were more likely to have decayed teeth and poor self-reported oral health [32]. The development of dental anxiety is most likely due to the anticipation of pain during dental procedures, being treated by dentist with indifferent demeanour, and concerns about actions undertaken by the dentist [49]. In this case, symptomatic attendance pattern is likely to result in more traumatic treatments, such as tooth extractions, which further exacerbate dental fear [50].

In relation to life course models [51], the concept of ‘accumulation of risk’ model supports our review findings [29, 30, 34, 35]. According to this model, one adverse or protective experience linked to subsequent circumstances accumulates over an individual’s life span, consequently affecting oral health outcomes in later life [19]. For example, children with irregular dental attendance or those raised in disadvantaged socioeconomic circumstances are more likely to report dental anxiety, engage in smoking, and exhibit poor oral hygiene behaviour [18, 32]. These factors, in turn, increased the risk of dental caries experience and oral impacts. Notwithstanding the temporal relationships established in the longitudinal studies, it remains plausible that there are common predictors influencing both dental visiting patterns and oral health outcomes, such as socioeconomic status, healthcare accessibility and dental anxiety. Another important finding that is worth highlighting is the possibility of a reciprocal interaction between dental visiting patterns and oral health. Although this interaction was not explicitly investigated in the papers included in this review, Åstrøm et al. [35] identified a reciprocal (bidirectional) interaction between long-term dental attendance and persistent tooth loss, both acting as predictors of OHRQoL. Previous studies have explored the impact of oral heath status on dental attendance, for instance, a longitudinal study conducted among Finnish adults found that poor OHRQoL led to nonregular dental service utilisation [52]. Additionally, a cross-sectional study involving the elderly population in Brazil discovered a positive association between having teeth and regular use of oral health services [53].

While this systematic review provides valuable insights, there are some limitations to be noted. First, of the 11 included papers, eight papers were derived from the same longitudinal study datasets, namely the Swedish and the Dunedin studies. Hence, there may be redundancy of data that potentially exaggerates the overall impression of the evidence. Second, a methodological concern is noted in one of the included papers from the Swedish study when ‘change in OIDP score’ was measured as the outcome [35]. The simultaneous improvement and deterioration in the OIDP scores among nonroutine attenders could be attributed to a potential ‘floor effect’. In this instance, it means that nonroutine attenders might exhibit a greater likelihood of experiencing either significant improvement or decline in OIDP scores than routine attenders. Third, variations in the categorisation of dental visiting patterns, outcome measures, range of age, follow‐up durations and selection of confounding variables contribute to an extensive heterogeneity in the data. Consequently, pooling of the results for meta-analysis was not feasible.

The included longitudinal studies demonstrate notable strengths, featuring prospective research design and remarkable follow-up durations, mostly spanning over a decade. However, several additional limitations relating to its context and practicalities should be acknowledged. First, all five longitudinal studies were from upper-middle- and high-income countries, with well-established dental services and adequate resources. This may limit the generalisability of the findings to broader populations in different settings, as there are apparent inequalities in dental workforce and oral healthcare utilisation between high-, middle- and lower-income countries [54, 55]. Moreover, studies in this review are from countries that have the lowest burden of untreated caries and severe periodontitis, as well as having a decreasing trend for tooth loss [1]. Therefore, subgroup differences from lower-middle- and lower-income countries, which could potentially influence oral health outcomes, may not be adequately addressed. Second, the majority of dental attendance data and oral health outcomes in the longitudinal studies relied on self-reporting, introducing potential recall bias, response bias and a degree of inaccuracy in exposure and outcome measurements [56]. Third, non-response or loss to follow-up, which is common in longitudinal studies, may introduce selection bias and questionable external validity of the results [38, 39, 56]. Respondents are more likely to be individuals who are health-conscious and inclined to attend follow-up appointments due to their intrinsic motivation for maintaining and enhancing their well-being, which distinguishes them from the non-respondents [28, 33, 35, 46].

Future research should consider two other important covariates: dental anxiety and chronic conditions. Despite ample evidence indicating their associations with dental attendance and oral health outcomes [49, 50, 57–60], only one paper in this review considered dental anxiety [32], and none considered chronic conditions. Furthermore, there is a need for longitudinal research and scientific evidence from different settings, particularly in lower- and lower-middle-income countries, which represent about 65% of the world population [61]. There are higher prevalence of oral diseases and a greater unmet need for dental services in lower- and middle-income countries than in other regions [1]; yet, they are proportionally less represented in research and publications, particularly low-income countries [62]. Finally, conducting analyses of this nature is unusual due to the lengthy follow-up period, limited resources and availability of oral health-related birth cohort studies. Improved routine data collection and enhanced compatibility for seamless data integration within the oral healthcare system across various sectors and institutions could pave the way for more extensive research and comprehensive evaluations in the field of dentistry. In the context of epidemiological research, the potential impact of big data linkage at the population level or integration of medical and dental could mark a transformative development, given the common data components shared between dentistry and medicine [63]. Data linkage not only has the potential to enable long-term monitoring of health outcomes but also to enhance seamless provision of patient care, support holistic health interventions, and facilitate the monitoring of rare diseases and healthcare expenses [64, 65].

## Conclusion

This systematic review of longitudinal studies identifies a significant association between dental visiting patterns and oral health in contexts where dental services are established. The data involve populations across the life course from five countries that were tracked over a span of up to two decades. The findings suggest that dental attendance serves as an important predictor of oral health, offering sufficient evidence to support the practice of encouraging routine dental checks in children and adults as outlined in the NICE guideline. This review could provide valuable evidence for early interventions and promotional strategies designed to prevent oral diseases in support of the WHO global strategy for oral health.

## Supplementary information

Supplementary File 1

Supplementary Table 1

Supplementary Table 2

## Data Availability

The data supporting the findings of this study are available within the article and its supplementary materials.
