# Supplementary material for: Impact of dental visiting patterns on oral health: A systematic review of longitudinal studies
Source: BDJ Open. 2024 Mar 6;10:18. doi: 10.1038/s41405-024-00195-7 (PMC10917741; doi:10.1038/s41405-024-00195-7)
Supplement: Supplementary file 1 [file 41405_2024_195_MOESM1_ESM.pdf]

File S1. Search strategy

Search concepts

- P-Population: Person’s life course, lifelong, lifetime, trajectory.
- E-Exposure: Dental visit, dental utilisation, dental care, oral healthcare, dental services, dental treatment.
- O-Outcome: Oral health, oral disease, OH-QoL, dental pain, toothache, dental caries, gingivitis, periodontitis, tooth loss.

Database: Ovid MEDLINE(R) ALL <1946 to March 02, 2023>

Search Strategy:

1 exp dental care/ (34596)

2 exp dental health services/ (39738)

3 ((oral or dental) adj2 (care or visit* or treatment* or healthcare or utili* or service* or appointment* or checkup* or check-up*)).mp. (94567)

4 1 or 2 or 3 (94567)

5 exp oral health/ (19961)

6 exp mouth diseases/ (327002)

7 exp dental caries/ (49860)

8 exp periodontal diseases/ (94678)

9 exp periodontitis/ (34311)

10 exp apical periodontitis/ (5353)

11 exp chronic periodontitis/ (3651)

12 exp gingivitis/ (11988)

13 exp toothache/ (2906)

14 exp tooth loss/ (4406)

15 ((dental or oral) adj5 health).mp. (61074)

16 ((mouth or oral or dental) adj3 disease*).mp. (48622)

17 caries.mp. (66325)

18 (carious adj3 (denti* or lesion* or teeth or tooth)).mp. (6096)

19 ((dental or tooth or teeth or root* or early) adj3 (caries or decay or cavit* or demineralі*)).mp. (81256)

20 ((periodontal or gum) adj3 disease*).mp. (43520)

21 periodontitis.mp. (41851)

22 ((apical or periapical or chronic or adult) adj3 periodontitis).mp. (13730)

23 gingivitis.mp. (15116)

24 toothache*.mp. (3917)

25 tooth loss.mp. (7915)

26 5 or 6 or 7 or 8 or 9 or 10 or 11 or 12 or 13 or 14 or 15 or 16 or 17 or 18 or 19 or 20 or 21 or 22 or 23 or 24 or 25 (476630)

27 exp longitudinal studies/ (163498)

28 exp cohort studies/ or exp case control study/ (2699588)

29 exp follow-up studies/ (690140)

30 exp prospective studies/ (1716376)

31 exp epidemiologic studies/ (3087159)

32 ((longitudinal or observational or cohort or concurrent or incidence or follow up or followup or follow-up or prospective or case-control) adj2 (stud* or analys* or survey* or design* or research)).mp. (3218873)

33 ((birth or close* or histor*) adj2 stud*).mp. (27063)

34 27 or 28 or 29 or 30 or 31 or 32 or 33 (3590551)

35 life course.mp. or exp Life Change Events/ (33878)

36 lifelong.mp. (23691)

37 lifetime.mp. (102570)

38 trajector*.mp. (102132)

39 35 or 36 or 37 or 38 (256724)

40 4 and 26 and 34 and 39 (130)

Database: Embase <1974 to 2023 March 02>

Search Strategy:

1 exp dental care/ (194609)

2 exp dental health services/ (194609)

3 ((oral or dental) adj2 (care or visit* or treatment* or healthcare or utili* or service* or appointment* or checkup* or check-up*)).mp. (126480)

4 1 or 2 or 3 (289924)

5 exp oral health/ (866817)

6 exp mouth diseases/ (626599)

7 exp dental caries/ (54209)

8 exp periodontal diseases/ (115021)

9 exp periodontitis/ (51675)

10 exp chronic periodontitis/ (5512)

11 exp gingivitis/ (18918)

12 exp toothache/ (8650)

13 exp tooth loss/ (115021)

14 ((dental or oral) adj5 health).mp. (55274)

15 ((mouth or oral or dental) adj3 disease*).mp. (57606)

16 caries.mp. (65443)

17 (carious adj3 (denti* or lesion* or teeth or tooth)).mp. (5937)

18 ((dental or tooth or teeth or root* or early) adj3 (caries or decay or cavit* or demineral i*)).mp. (66185)

19 ((periodontal or gum) adj3 disease*).mp. (62909)

20 periodontitis.mp. (47761)

21 ((apical or periapical or chronic or adult) adj3 periodontitis).mp. (13332)

22 gingivitis.mp. (20996)

23 toothache*.mp. (2167)

24 tooth loss.mp. (6385)

25 5 or 6 or 7 or 8 or 9 or 10 or 11 or 12 or 13 or 14 or 15 or 16 or 17 or 18 or 19 or 20 or 21 or 22 or 23 or 24 (1524363)

26 exp longitudinal studies/ (189535)

27 exp cohort studies/ or exp case control study/ (1199050)

28 exp follow-up studies/ (2011288)

29 exp prospective studies/ (2220725)

30 exp epidemiologic studies/ (4382092)

31 ((longitudinal or observational or cohort or concurrent or incidence or follow up or followup or follow-up or prospective or case-control) adj2 (stud* or analys* or survey* or design* or research)).mp. (3784433)

32 ((birth or close* or histor*) adj2 stud*).mp. (38374)

33 26 or 27 or 28 or 29 or 30 or 31 or 32 (7911500)

34 life course.mp. or exp Life Change Events/ (43668)

35 lifelong.mp. (34613)

36 lifetime.mp. (125379)

37 trajector*.mp. (126330)

38 34 or 35 or 36 or 37 (322941)

39 4 and 25 and 33 and 38 (362)

Web of Science Search Strategy (v0.1)

Database: All Databases

Entitlements:

- WOS: 1900 to 2023
- KJD: 1980 to 2023 (Korean Journal Database)
- MEDLINE: 1950 to 2023
- PPRN: 1991 to 2023 (Preprint)
- SCIELO: 2002 to 2023

Searches:

1: TS=((oral or dental) NEAR/2 (care or visit* or treatment* or healthcare or utili* or service* or appointment* or checkup* or check-up*)) and Preprint Citation Index (Exclude – Database)

Date Run: Thu Mar 02 2023 11:42:49 GMT+0000 (Greenwich Mean Time)

Results: 130472

2: TS=((dental or oral) NEAR/5 health) and Preprint Citation Index (Exclude – Database)

Date Run: Thu Mar 02 2023 11:43:39 GMT+0000 (Greenwich Mean Time)

Results: 83664

3: TS=((mouth or oral or dental) NEAR/3 disease*) and Preprint Citation Index (Exclude – Database)

Date Run: Thu Mar 02 2023 11:44:06 GMT+0000 (Greenwich Mean Time)

Results: 196341

4: TS=(caries) and Preprint Citation Index (Exclude – Database)
Date Run: Thu Mar 02 2023 11:44:21 GMT+0000 (Greenwich Mean Time)
Results: 91137

5: TS=(carious NEAR/3 (denti* or lesion* or teeth or tooth)) and Preprint Citation Index (Exclude – Database)
Date Run: Thu Mar 02 2023 11:44:30 GMT+0000 (Greenwich Mean Time)
Results: 9181

6: TS=((dental or tooth or teeth or root* or early) NEAR/3 (caries or decay or cavit* or demineral i*)) and Preprint Citation Index (Exclude – Database)
Date Run: Thu Mar 02 2023 11:44:41 GMT+0000 (Greenwich Mean Time)
Results: 103886

7: TS=((periodontal or gum) NEAR/3 disease*) and Preprint Citation Index (Exclude – Database)
Date Run: Thu Mar 02 2023 11:44:53 GMT+0000 (Greenwich Mean Time)
Results: 62051

8: TS=(periodontitis) and Preprint Citation Index (Exclude – Database)
Date Run: Thu Mar 02 2023 11:45:04 GMT+0000 (Greenwich Mean Time)
Results: 55337

9: TS=((apical or periapical or chronic or adult) NEAR/3 periodontitis) and Preprint Citation Index (Exclude – Database)
Date Run: Thu Mar 02 2023 11:45:12 GMT+0000 (Greenwich Mean Time)
Results: 18946

10: TS=(gingivitis) and Preprint Citation Index (Exclude – Database)
Date Run: Thu Mar 02 2023 11:45:22 GMT+0000 (Greenwich Mean Time)
Results: 20047

11: TS=(toothache*) and Preprint Citation Index (Exclude – Database)
Date Run: Thu Mar 02 2023 11:45:31 GMT+0000 (Greenwich Mean Time)
Results: 4704

12: TS=(tooth NEAR/3 loss) and Preprint Citation Index (Exclude – Database)
Date Run: Thu Mar 02 2023 11:47:11 GMT+0000 (Greenwich Mean Time)
Results: 15795

13: #12 OR #11 OR #10 OR #9 OR #8 OR #7 OR #6 OR #5 OR #4 OR #3 OR #2 OR #1 and Preprint Citation Index (Exclude – Database)
Date Run: Thu Mar 02 2023 11:47:23 GMT+0000 (Greenwich Mean Time)
Results: 503495

14: TS=((longitudinal or observational or cohort or concurrent or incidence or follow up or prospective or case control or case-control) (stud* or analys* or survey* or design* or research)) and Preprint Citation Index (Exclude – Database)

Date Run: Thu Mar 02 2023 11:51:21 GMT+0000 (Greenwich Mean Time)

Results: 6720229

15: TS=((birth or close* or histor*) NEAR/2 stud*) and Preprint Citation Index (Exclude – Database)

Date Run: Thu Mar 02 2023 11:51:35 GMT+0000 (Greenwich Mean Time)

Results: 119734

16: #15 OR #14 and Preprint Citation Index (Exclude – Database)

Date Run: Thu Mar 02 2023 11:51:57 GMT+0000 (Greenwich Mean Time)

Results: 6806556

17: TS=(life course) and Preprint Citation Index (Exclude – Database)

Date Run: Thu Mar 02 2023 11:52:07 GMT+0000 (Greenwich Mean Time)

Results: 139485

18: TS=(lifelong) and Preprint Citation Index (Exclude – Database)

Date Run: Thu Mar 02 2023 11:52:13 GMT+0000 (Greenwich Mean Time)

Results: 44093

19: TS=(lifetime) and Preprint Citation Index (Exclude – Database)

Date Run: Thu Mar 02 2023 11:52:19 GMT+0000 (Greenwich Mean Time)

Results: 442394

20: TS=(trajector*) and Preprint Citation Index (Exclude – Database)

Date Run: Thu Mar 02 2023 11:52:25 GMT+0000 (Greenwich Mean Time)

Results: 406850

21: #20 OR #19 OR #18 OR #17 and Preprint Citation Index (Exclude – Database)

Date Run: Thu Mar 02 2023 11:52:35 GMT+0000 (Greenwich Mean Time)

Results: 1018065

22: #21 AND #16 AND #13 and Preprint Citation Index (Exclude – Database)

Date Run: Thu Mar 02 2023 11:54:43 GMT+0000 (Greenwich Mean Time)

Results: 1390

CINAHL

Thursday, March 02, 2023 12:15:21 PM

S6	S1 AND S2 AND S3 AND S4	Expanders<br>- Apply equivalent subjects<br>Search modes<br>-Boolean/Phrase	Interface<br>- EBSCO host Research Databases<br>Search Screen<br>- Advanced Search<br>Database<br>- CINAHL	86
S5	TX (cross sectional study or cross-sectional study) OR TX cross-sectional research OR TX cross sectional OR TX (case report or case study or clinical case or case series) OR TX (meta-analysis or systematic review or literature review or metaanalysis or overview or review or meta-synthesis or metasynthesis)	Expanders<br>- Apply equivalent subjects<br>Search modes<br>-Boolean/Phrase	Interface<br>- EBSCO host Research Databases<br>Search Screen<br>- Advanced Search<br>Database<br>- CINAHL	2,034,611
S4	TX (life course theory or life course perspective or life course approach) OR TX lifespan OR TX lifetime	Expanders<br>- Apply equivalent subjects	Interface<br>- EBSCO host Research Databases<br>Search Screen	76,023

	OR TX lifelong OR TX (trajectory or trajectories) OR TX trajectory theory OR TX trajectory model	Search modes -Boolean/Phrase	- Advanced Search Database<br>- CINAHL	
S3	TX (longitudinal studies or longitudinal research or longitudinal method) OR TX (cohort study or cohort) OR TX (epidemiology orincidence or prevalence or occurrence) OR TX cohort epidemiology study OR TX birth cohort OR TX prospective study OR TX follow-up studies OR TX(observational study or observational research or observational method) OR TX (case control study or case-control)	Expanders<br>- Apply equivalent subjects<br>Search modes<br>-Boolean/Phrase	Interface<br>- EBSCO host Research Databases<br>Search Screen<br>- Advanced Search Database<br>- CINAHL	1,607,829
S2	TX (dental health or oral health or dental hygiene or oral hygiene) OR TX mouth diseases OR TX oral disease OR TX (dental caries or dental decay or dental cavity or dental cavities or tooth decay) OR TX (periodontal disease or periodontitis or chronic periodontitis or gingivitis) OR TX toothache OR TX tooth loss OR TX oral health-related quality of life	Expanders<br>- Apply equivalent subjects<br>Search modes<br>-Boolean/Phrase	Interface<br>- EBSCO host Research Databases<br>Search Screen<br>- Advanced Search Database<br>- CINAHL	36,129
S1	TX (dental care or oral care) OR TX dental visits OR TX dental services OR TX dental service utilization OR TX dental service need OR TX dental appointment OR TX dental check up	Expanders<br>- Apply equivalent subjects<br>Search modes<br>-Boolean/Phrase	Interface<br>- EBSCO host Research Databases<br>Search Screen<br>- Advanced Search Database<br>- CINAHL	53,397

SCOPUS

EXPORT DATE:02 Mar 2023 RESULTS: 304

ALL ("oral care" OR "oral visit*" OR "oral treatment*" OR "oral healthcare" OR "oral utili*" OR "oral service*" OR "oral appointment*" OR "oral check up" OR "dental care"

OR "dental visit*" OR "dental treatment*" OR "dental healthcare" OR "dental utili*" OR "dental service*" OR "dental appointment*" OR "dental check up") AND ALL ("dental health" OR "oral health" OR "oral health related quality of life" OR "mouth disease*" OR "dental disease*" OR "oral disease*" OR caries OR "carious denti*" OR "carious teeth" OR "carious tooth" OR "carious lesion*" OR "dental caries" OR "dental decay" OR "dental cavit*" OR "dental deminerali*" OR "t??th caries" OR "t??th decay" OR "t??th cavit*" OR "t??th deminerali*" OR "root caries" OR "root decay" OR "root cavit*" OR "root deminerali*" OR "early caries" OR "early decay" OR "early cavit*" OR "early deminerali*" OR "periodontal disease*" OR "gum disease*" OR periodontitis OR "apical periodontitis" OR "periapical periodontitis" OR "chronic periodontitis" OR "adult periodontitis" OR gingivitis OR toothache* OR "tooth loss") AND ALL ("longitudinal stud*" OR "longitudinal analys*" OR "longitudinal survey*" OR "longitudinal design*" OR "longitudinal research" OR "observational stud*" OR "observational analys*" OR "observational survey*" OR "observational design*" OR "observational research" OR "cohort stud*" OR "cohort analys*" OR "cohort survey*" OR "cohort design*" OR "concurrent stud*" OR "concurrent analys*" OR "concurrent survey*" OR "incidence stud*" OR "incidence analys*" OR "incidence survey*" OR "follow up stud*" OR "follow up analys*" OR "follow up survey*" OR "prospective stud*" OR "prospective analys*" OR "prospective survey*" OR "case control stud*" OR "case control analys*" OR "case-control stud*" OR "case-control analys*" OR "birth cohort stud*" OR "close cohort stud*") AND ALL ("life course" OR "lifelong" OR "lifetime" OR "trajector*") AND NOT ALL ("cross-sectional stud*" OR "cross-sectional analys*" OR "cross sectional stud*" OR "cross sectional analys*" OR "meta analys*" OR "systematic review*" OR "case report*" OR "case stud*")
