# Supplementary material for: Impact of dental visiting patterns on oral health: A systematic review of longitudinal studies
Source: BDJ Open. 2024 Mar 6;10:18. doi: 10.1038/s41405-024-00195-7 (PMC10917741; doi:10.1038/s41405-024-00195-7)
Supplement: Supplementary file 2 — Supplementary Table 1 [file 41405_2024_195_MOESM2_ESM.pdf]

Table S1. The Newcastle-Ottawa Scale (NOS) quality assessment for cohort study.

**Components**	**Response options**
**Selection**	
**1. Representativeness of the exposed cohort**	
a) truly representative of the average exposed individuals in the community.	☆
b) somewhat representative of the average exposed individuals in the community.	☆
c) selected group of users only, e.g., nurses, volunteers.	/
d) no description of the derivation of the cohort.	/
**2. Selection of the non-exposed cohort**	
a) drawn from the same community as the exposed cohort.	☆
b) drawn from a different source.	/
c) no description of the derivation of the non-exposed cohort.	/
**3. Ascertainment of exposure**	
a) secure record (eg surgical records)	☆
b) structured interview	☆
c) written self-report	/
d) no description	/
**4. Demonstration that outcome of interest was not present at start of study**	
a) yes	☆
b) no	/
**Comparability***	
**5. Comparability of cohorts on the basis of the design or analysis**	
a) study controls for age, sex, SES (ethnicity/occupation/income/education/social class).	☆
b) study controls for any additional factor - dental anxiety, healthcare access/cost.	☆

Components	Response options
**Outcome**	
**6. Assessment of outcome**	
a) independent blind assessment	☆
b) record linkage	☆
c) self-report	/
d) no description	/
**7. Was follow-up long enough for outcomes to occur**	
a) yes (≥ 2years)	☆
b) no	/
**8. Adequacy of follow up of cohorts**	
a) complete follow up - all subjects accounted for.	☆
b) subjects lost to follow up unlikely to introduce bias - small number lost (<20%), or description provided of those lost.	☆
c) follow up rate < 80% and no description of those lost.	/
d) no statement.	/
NOTE: A study can be awarded a maximum of **one star** for **each numbered item within the Selection and Exposure** categories; however, *maximum of **two stars can be given for Comparability**.	

Wells GA, Shea B, O'Connell D, Peterson J, Welch V, Losos M, et al. The Newcastle-Ottawa Scale (NOS) for Assessing the Quality of Nonrandomized Studies in Meta-Analyses: Ottawa Hospital Research Institute; 2021 [2 February 2023]. Available from: https://www.ohri.ca/programs/clinical_epidemiology/oxford.asp.
