# Supplementary material for: Impact of dental visiting patterns on oral health: A systematic review of longitudinal studies
Source: BDJ Open. 2024 Mar 6;10:18. doi: 10.1038/s41405-024-00195-7 (PMC10917741; doi:10.1038/s41405-024-00195-7)
Supplement: Supplementary file 3 — Supplementary Table 2 [file 41405_2024_195_MOESM3_ESM.pdf]

Table S2. Characteristics of included papers.

Paper	Study name	Study population	Sample size	Follow-up period	Study methods	Exposure groups	Primary outcomes	Measures	Results
Åstrøm *et al.* (2011)[28]	1942 Sweden birth cohort study	Sweden; Adults aged 50 years (at year 1992) from two counties of Sweden	n=4143 Male: 47.8% Female: 52.2%	15 years	- Involved 4 sweeps, at ages of 50, 55, 60 and 65.<br>- Self-administered questionnaires.<br>- Secondary data analysis.	Dental attendance at ages 50 & 65.<br>- stable annual<br>- annual - not annual (downward)<br>- not annual - annual (upward)<br>- stable not annual	OHRQoL using 8-item OIDP at 65 years old.	ORs	<u>OIDP (adjusted)</u><br>- annual-not annual vs stable annual, OR=1.3 (95% CI 1.0-1.6)<br>- not annual-annual vs stable annual, NS<br>- stable not annual vs stable annual, NS
Åstrøm *et al.* (2011)[29]	1942 Sweden birth cohort study	Sweden; Adults aged 50 years (at year 1992) from two counties of Sweden	n=4143 Male: 47.8% Female: 52.2%	15 years	- Involved 4 sweeps, at ages of 50, 55, 60 and 65.<br>- Self-administered questionnaires.<br>- Secondary data analysis.	Dental care utilisation at 60 years old.<br>- Yes<br>- No	Tooth loss patterns between age 50 and 65 years old.	ORs	<u>Tooth loss across adulthood life course (50-55-60-65 years old)</u><br>- In adjusted model, dental care vs no dental care = NS<br><u>Tooth loss between 50 and 65 years old</u>.<br>- In adjusted model, dental care vs no dental care = NS
Astrøm *et al.* (2014)[33]	1942 Sweden birth cohort study	Sweden; Adults aged 50 years (at year 1992) from two counties of Sweden	n=4143 Male: 47.8% Female: 52.2%	15 years	- Involved 4 sweeps, at ages of 50, 55, 60 and 65.<br>- Self-administered questionnaires.<br>- Secondary data analysis.	Routine dental care utilisation at '92 (50 years old) and '07 (65 years old)<br>(0) nonroutine '92&'07<br>(1) routine '92 - nonroutine '07<br>(2) nonroutine '92 - routine '07<br>(3) routine '92&'07	At age 65 (2007),<br>- OHRQoL using 8-item OIDP<br>- Dentition status (major tooth loss 'lost many or all teeth')	ORs	<u>OIDP</u> (adjusted):<br>- routine '92 - nonroutine '07 vs nonroutine '92&'07, NS<br>- nonroutine '92 - routine '07 vs nonroutine '92&'07, OR=0.4 (95% CI 0.3-0.5)<br>- routine '92/'07 vs nonroutine '92&'07, OR=0.3 (95% CI 0.2-0.5)<br><u>Major tooth loss</u> (adjusted):<br>- routine '92 - nonroutine '07 vs nonroutine '92&'07, NS<br>- nonroutine '92 - routine '07 vs nonroutine '92&'07, OR=0.8 (CI 0.6-0.9)<br>- routine '92&'07 vs nonroutine '92&'07, OR=0.6 (95% CI 0.4-0.7)

Paper	Study name	Study population	Sample size	Follow-up period	Study methods	Exposure groups	Primary outcomes	Measures	Results
Astrøm *et al.* (2018)[35]	1942 Sweden birth cohort study	Sweden; Adults aged 50 years (at year 1992) from two counties of Sweden	n=3585 Male: 47.6% Female: 52.4%	20 years	- Involved 5 sweeps, at ages of 50, 55, 60, 65 and 70.<br>- Self-administered questionnaires.<br>- Secondary data analysis.	Long-term routine dental attendance at '92 (50 years old) and '07 (65 years old)<br>(0) nonroutine '92&'07<br>(1) routine '92 - nonroutine '07<br>(2) nonroutine '92 - routine '07<br>(3) routine '92&'07<br><br>Long-term attendance at specialist dental care at '07 (65 years old) and '12 (70 years old).<br>(0) no specialist care attendance '92&'07<br>(1) specialist care attendance '92/'07<br>(2) specialist attendance '92&'07	Change in OIDP scores between '07 (65 years old) and '12 (70 years old)	ORs	<u>Dental attendance patterns on worsened OIDP (adjusted)</u><br>- routine'92-nonroutine'07 vs nonroutine '92&'07, NS<br>- nonroutine'92-routine'07 vs nonroutine '92&'07, OR=0.6 (95% CI 0.4-0.9)<br>- routine '92&'07 vs nonroutine '92&'07, OR=0.4 (95% CI 0.4-0.8)<br><u>Dental attendance on improved OIDP (adjusted)</u><br>- routine'92-nonroutine'07 vs nonroutine '92&'07, NS<br>- nonroutine'92-routine'07 vs nonroutine '92&'07, OR=0.5 (95% CI 0.4-0.7)<br>- routine '92&'07 vs nonroutine '92&'07, OR=0.4 (95% CI 0.3-0.6)<br><u>Specialist attendance care on worsened OIDP (adjusted)</u><br>- specialist care '92/'07 vs no specialist care '92&'07, NS<br>- specialist care '92&'07 vs no specialist care '92&'07, OR=1.4 (95% CI 1.1-1.8)<br><u>Specialist attendance care on improved OIDP (adjusted)</u><br>- specialist care '92/'07 vs no specialist care '92&'07, OR=1.6 (95% CI 1.3-2.1)<br>- specialist care '92&'07 vs no specialist care '92&'07, OR=1.6 (95% CI 1.2-2.0)

Paper	Study name	Study population	Sample size	Follow-up period	Study methods	Exposure groups	Primary outcomes	Measures	Results
Thomson *et al.* (2010)[18]	Dunedin Multidisciplinary Health and Development Study (birth cohort study)	New Zealand; Study members born between April 1972 and March 1973 in Dunedin	n=932<br>Male: 51.1%<br>Female: 48.9%	17 years	- Involved 4 sweeps at ages 15, 18, 26 and 32.<br>- Interview and clinical assessment.<br>- Secondary data analysis.	Use of dental services at ages 15, 18, 26 and 32.<br>- routine attenders<br>- nonroutine attenders	At age 32,<br>- mean DMFS<br>- mean D<br>- prevalence of 1+ teeth missing due to dental caries<br>- self-reported oral health	ORs and IRRs	Self-rated OH 'better than average' (adjusted),<br>- Age 15: routine vs nonroutine, OR=1.70 (95% CI 1.16-2.50)<br>- Age 18: routine vs nonroutine, OR=1.52 (95% CI 1.12-2.05)<br>- Age 26: routine vs nonroutine, OR=2.24 (95% CI 1.65-3.03)<br>- Age 32: routine vs nonroutine, OR=3.36 (95% CI 2.42-4.66)<br>1+ teeth missing due to dental caries (adjusted),<br>- Age 15: routine vs nonroutine, NS<br>- Age 18: routine vs nonroutine, OR=0.66 (95% CI 0.46-0.95)<br>- Age 26: routine vs nonroutine, OR=0.35 (95% CI 0.23-0.54)<br>- Age 32: routine vs nonroutine, OR=0.54 (95% CI 0.36-0.82)<br>Mean DS (adjusted),<br>- Age 15: routine vs nonroutine, IRR=0.64 (95% CI 0.42-0.96)<br>- Age 18: routine vs nonroutine, IRR=0.67 (95% CI 0.49-0.90)<br>- Age 26: routine vs nonroutine, IRR=0.60 (95% CI 0.43-0.84)<br>- Age 32: routine vs nonroutine, IRR=0.54 (95% CI 0.40-0.73)<br>Mean DMFS (adjusted),<br>- Age 15: routine vs nonroutine, NS<br>- Age 18: routine vs nonroutine, IRR=0.83 (95% CI 0.73-0.95)<br>- Age 26: routine vs nonroutine, IRR=0.86 (95% CI 0.75-0.98)<br>- Age 32: routine vs nonroutine, NS

Paper	Study name	Study population	Sample size	Follow-up period	Study methods	Exposure groups	Primary outcomes	Measures	Results
Crocombe *et al.* (2012)[32]	Dunedin Multidisciplinary Health and Development Study (birth cohort study)	New Zealand; Study members born between April 1972 and March 1973 in Dunedin	n=833 sex proportion not stated	17 years	- Involved 4 sweeps at ages 15, 18, 26 and 32.<br>- Interview and clinical assessment.<br>- Secondary data analysis.	Dental visiting trajectories at ages 15, 18, 26 and 32.<br>- regular attenders<br>- decliners<br>- opportunists	Oral health at age 32<br>-mean DMFS<br>-mean D, M, F (separately)<br>-mean OHIP-14 score<br>- self-rated OH: dichotomised into 'better than average' and 'worse than average'	RRs and coefficient (β) values	<u>DMFS (adjusted)</u><br>- opportunist vs regular, β =3.9 (95% CI 0.2-7.6)<br>- decliner vs regular, NS<br><u>Missing (adjusted)</u><br>- opportunist vs regular β =0.7 (95% CI 0.2-1.2)<br>- decliner vs regular, NS<br><u>Filled (adjusted)</u><br>- opportunist vs regular, NS<br>- decliner vs regular, NS<br><u>Decayed (adjusted)</u><br>- opportunist vs regular, NS<br>- decliner vs regular, NS<br><u>OHIP-14 (adjusted)</u><br>- opportunist vs regular, β =2.1 (95% CI 0.4-3.8)<br>- decliner vs regular, β =2.1 (95% CI 0.9-3.2)<br><u>Self-rated OH (adjusted)</u><br>- opportunist vs regular, RR=0.8 (95% CI 0.7-0.9)<br>- decliner vs regular, RR=0.8 (95% CI 0.7-0.9)
Broadbent *et al.* (2016)[34]	Dunedin Multidisciplinary Health and Development Study (birth cohort study)	New Zealand; Study members born between April 1972 and March 1973 in Dunedin	n=848 sex proportion not stated	12 years	- Involved 3 sweeps at ages 26, 32, and 38.<br>- Interview and clinical assessment.<br>- Secondary data analysis.	Dental attendance at ages 26 and 32.<br>- regular<br>- nonregular	Dental caries experience (count) at age 38:<br>- decayed tooth surfaces<br>- missing tooth surfaces	Standard estimate values	Dental attendance → decayed tooth surfaces<br>Std estimate= -1.184 (95% CI -2.286 to -0.395)<br><br>Dental attendance → missing tooth surfaces<br>Estimate= -5.883 (95% CI -10.33 to -2.811)

Paper	Study name	Study population	Sample size	Follow-up period	Study methods	Exposure groups	Primary outcomes	Measures	Results
Hong *et al.* (2023)[37]	Dunedin Multidisciplinary Health and Development Study (birth cohort study)	New Zealand; Study members born between April 1972 and March 1973 in Dunedin	n=844 sex proportion not stated	19 years	- Involved 4 sweeps at ages of 26, 32, 38 and 45.<br>- Interview.<br>- Secondary data analysis.	Dental visiting patterns at age 26, 32, 38 and 45.<br>- never routine<br>- sometimes routine<br>- always routine	Change in mean OHIP-14 scores between age 32 and 45.<br>Change in prevalence of OHIP-14 impacts at age 32 and 45.	IRRs and ORs	<u>Change in OHIP-14 scores (proportion)</u><br>- Worsened: never > sometimes > always routine<br>- Stable no change: always > sometimes > never routine<br>- Improved: always > never > sometimes<br><u>Mean OHIP-14 scores (adjusted)</u><br>- sometimes vs never routine attenders, IRR=0.85 (95% CI 0.76-0.94)<br>- always vs never routine attenders, IRR=0.59 (95% CI 0.44-0.80)<br><u>OHIP-14 impact prevalence (adjusted)</u><br>- sometimes vs never routine attenders, OR=0.70 (95% CI 0.54-0.92)<br>- always vs never routine attenders, NS
Crocombe *et al.* (2012)[31]	Australian study	Australia; Tasmanian aged ≥15years	n=362<br>Male: 50.8%<br>Female: 49.2%	12 months	- 12-month follow-up by mail self-complete questionnaire, dental attendance from the service-use logbook or the dental treatment audit.<br>- Primary data analysis	Dental attendance<br>- Yes/No<br><br>Patterns of attendance at baseline<br>- regularity: ≤12 months / >12 months<br>- usual reason: check-up / dental problem	Change in OHIP-14 severity	Estimated values	<u>Change in OHIP-14 severity</u><br>- Dental attendance was associated with change in mean OHIP-14 severity (estimate=1.73, SE=0.66, p-value=0.01).<br>- No significant association between patterns of attendance and change in OHIP-14 severity (from bivariate analysis)

Paper	Study name	Study population	Sample size	Follow-up period	Study methods	Exposure groups	Primary outcomes	Measures	Results
Lu *et al.* (2011)[30]	Hong Kong study	Hong Kong, China; Secondary schoolchildren aged 12 years	n= 232 Male: 52.2% Female: 47.8%	6 years	- Self-completed questionnaires and clinical examination at three time points: 12, 15 and 18 years old.<br>- Primary data analysis	Utilisation of dental services during adolescence<br>-Yes/No	DMFT index and periodontal health status at age 12, 15, 18.<br>- mean DMFT value<br>- highest CPI score	coefficient (β) values	- utilisation of dental service at 12-15years → utilisation of dental service at 15-18years (β =0.432, $p<0.05$)<br>- utilisation of dental service at 15-18years → mean DMFT (β =0.117, $p<0.05$)<br>- utilisation of dental service at any point of time → CPI score (NS)

Paper	Study name	Study population	Sample size	Follow-up period	Study methods	Exposure groups	Primary outcomes	Measures	Results
Menegazzo *et al.* (2020)[36]	Brazilian study	Brazil; Preschool children aged 12-59 months in Santa Maria	n=449<br>Male: 49.0%<br>Female: 51.0%	7years	- Self-completed questionnaires by parents for dental attendance; face-to-face interview with children for the CPQ8-10 (Brazilian version) at follow-up.<br>- Primary data analysis	Long-term routine dental attendance at baseline (T1) & f/up (T2)<br>(0) Routine T1 & T2<br>(1) Nonroutine attendance/non-users T1 & Routine T2<br>(2) Routine T1 & Nonroutine attendance/non-users T2<br>(3) Nonroutine attendance/non-users T1 & T2	Mean CPQ8-10 score at follow-up	IRRs	<u>Overall CPQ8-10 score (adjusted)</u><br>- (1) vs (0), IRR=2.20 (95% CI 1.67-2.91)<br>- (2) vs (0), IRR=1.70 (95% CI 1.28-2.25)<br>- (3) vs (0), IRR=2.05 (95% CI 1.59-2.66)<br>- Nonroutine T1 vs Routine T1, IRR=1.31 (95% CI 1.16-1.49)<br>- Nonroutine T2 vs Routine T2, NS<br><u>Domain 1: Oral symptoms</u><br>- (1) vs (0), IRR=1.58 (95% CI 1.09-2.28)<br>- (2) vs (0), NS<br>- (3) vs (0), IRR=1.42 (95% CI 1.02-1.98)<br>- Nonroutine T1 vs Routine T1, IRR=1.22 (95% CI 1.02-1.46)<br>- Nonroutine T2 vs Routine T2, NS<br><u>Domain 2: Functional limitation</u><br>- (1) vs (0), NS<br>- (2) vs (0), NS<br>- (3) vs (0), IRR=2.16 (95% CI 1.21-3.88)<br>- Nonroutine T1 vs Routine T1, IRR=1.58 (95% CI 1.18-2.12)<br>- Nonroutine T2 vs Routine T2, IRR=1.31 (95% CI 1.00-1.71)<br><u>Domain 3: Emotional well-being</u><br>- (1) vs (0), IRR=2.54 (95% CI 1.29-4.99)<br>- (2) vs (0), IRR=2.59 (95% CI 1.31-5.09)<br>- (3) vs (0), IRR=2.44 (95% CI 1.29-4.59)<br>- Nonroutine T1 vs Routine T1, NS<br>- Nonroutine T2 vs Routine T2, NS<br><u>Domain 4: Social well-being</u><br>- (1) vs (0), IRR=15.09 (95% CI 3.68-61.87)<br>- (2) vs (0), IRR=9.86 (95% CI 2.38-40.80)<br>- (3) vs (0), IRR=11.90 (95% CI 2.95-47.93)<br>- Nonroutine T1 vs Routine T1, IRR=1.60 (95% CI 1.18-2.17)<br>- Nonroutine T2 vs Routine T2, NS

OHRQoL: Oral Health-Related Quality of Life; OIDP: Oral Impacts on Daily Performance; OHIP: Oral Health Impact Profile; CPQ: Child Perception Questionnaire; CPI: Community Periodontal Index; DMFS: Decayed, Missing and Filled Surfaces; DMFT: Decayed, Missing and Filled Teeth; D: Decayed; M: Missing; F: Filled; OR: Odds Ratio; IRR: Incidence Rate Ratio; NS: Not Significant.
